# Neuroanatomical and neurocognitive changes associated with subjective cognitive decline

**DOI:** 10.3389/fmed.2023.1094799

**Published:** 2023-02-02

**Authors:** Miguel Ángel Rivas-Fernández, Mónica Lindín, Montserrat Zurrón, Fernando Díaz, Cristina Lojo-Seoane, Arturo X. Pereiro, Santiago Galdo-Álvarez

**Affiliations:** ^1^Department of Clinical Psychology and Psychobiology, Universidade de Santiago de Compostela, Santiago de Compostela, Spain; ^2^Cognitive Neuroscience Research Group, Health Research Institute of Santiago de Compostela (IDIS), Santiago de Compostela, Spain; ^3^Department of Developmental and Educational Psychology, Universidade de Santiago de Compostela, Santiago de Compostela, Spain

**Keywords:** Alzheimer’s disease, subjective cognitive decline (SCD), structural magnetic resonance imaging, brain structural changes, subjective cognitive complaints

## Abstract

**Introduction:**

Subjective Cognitive Decline (SCD) can progress to mild cognitive impairment (MCI) and Alzheimer’s disease (AD) dementia and thus may represent a preclinical stage of the AD continuum. However, evidence about structural changes observed in the brain during SCD remains inconsistent.

**Materials and methods:**

This cross-sectional study aimed to evaluate, in subjects recruited from the CompAS project, neurocognitive and neurostructural differences between a group of forty-nine control subjects and forty-nine individuals who met the diagnostic criteria for SCD and exhibited high levels of subjective cognitive complaints (SCCs). Structural magnetic resonance imaging was used to compare neuroanatomical differences in brain volume and cortical thickness between both groups.

**Results:**

Relative to the control group, the SCD group displayed structural changes involving frontal, parietal, and medial temporal lobe regions of critical importance in AD etiology and functionally related to several cognitive domains, including executive control, attention, memory, and language.

**Conclusion:**

Despite the absence of clinical deficits, SCD may constitute a preclinical entity with a similar (although subtle) pattern of neuroanatomical changes to that observed in individuals with amnestic MCI or AD dementia.

## 1. Introduction

The neuropathological onset of Alzheimer’s disease (AD), the most common cause of dementia, may occur several decades before the emergence of clinical symptoms. Regarding cognitive impairment, the following cognitive stages have been proposed: cognitively unimpaired (CU, corresponding to a control group), subjective cognitive decline (SCD), mild cognitive impairment (MCI), and AD dementia.

Within the AD continuum, SCD has been proposed as a possible preclinical stage that includes a subset of CU individuals with normal performance in standardized cognitive tests (adjusted for age, sex, and education), who report subjective cognitive complaints and who have an increased risk of future objective cognitive decline (1). SCD is characterized by two main criteria: (1) a self-experienced persistent decline in cognitive capacity, relative to a previously normal cognitive status, which is unrelated to an acute event; and (2) normal performance on standardized cognitive tests used to classify MCI, adjusted for age, sex, and education (1, 2). As the disease progresses, cognitive deficits arise and can lead to MCI, a syndrome in which cognitive impairment can be objectively measured by neuropsychological examinations (3), and daily life activities are preserved, although cognitive difficulty may have a mild functional impact on more complex activities of daily life (4).

In recent years, there has been growing interest in assessing the rate of conversion of people diagnosed with preclinical AD to dementia. It has been demonstrated that people with subjective memory complaints, but not objective impairment, are two times more likely to develop dementia than individuals without subjective memory complaints. The annual conversion rates in these individuals are 6.6% to MCI and 2.3% to dementia, compared with 1% in those without subjective memory complaints (5). Thus, the early detection of individuals at risk of converting to AD dementia will have important implications for the early prevention of cognitive impairment through the implementation of pharmacological and/or non-pharmacological interventions. SCD thus represents a pre-symptomatic stage of interest and in which it may be possible to identify early brain changes that emerge before the onset of clinical symptoms.

Neuroimaging techniques with high spatial resolution, such as structural magnetic resonance imaging (sMRI), enable accurate *in vivo* examination of subtle changes that may affect the brain structure of individuals with preclinical AD. Thus, Schwarz et al. (6) proposed the “AD signature index,” a neuroimaging biomarker that covers brain regions that are highly vulnerable to displaying neurodegenerative changes related to AD dementia. Assessment of the AD signature in possible preclinical stages such as SCD is of interest for examining the potential association between SCD and the development of AD dementia.

However, sMRI studies on SCD have reported inconsistent findings. Some studies have shown that individuals with SCD display a pattern of structural changes similar to those observed in subjects with amnestic MCI or AD dementia and involving medial temporal lobe (MTL, e.g., hippocampus, entorhinal cortex), frontal and posterior parietal regions (7–9), suggesting that SCD may represent a preclinical stage between normal aging and MCI. By contrast, other studies did not find any significant neuroanatomical differences between individuals with SCD and control subjects (10, 11), suggesting that microstructural changes in SCD may not be easy to detect.

These inconsistent findings have been attributed to several factors, including variations in study settings (community-recruited volunteers or participants from memory clinics), the use of different diagnostic criteria/methods of assessing SCD and differences in MRI strength and/or methodological approaches (e.g., voxel or surface based morphometry, manual or automatic segmentations) (12, 13).

Regarding the variations in study settings, it has been pointed out that, despite some common aspects, the pattern of neuroanatomical changes differs between SCD community-recruited volunteers and individuals with SCD who are recruited from memory clinics (13). Evidence from SCD-community samples indicates an AD-specific pattern of neurostructural changes involving MTL structures (e.g., hippocampus and entorhinal cortex) (14–17) as well as the temporo-parietal cortex (18–20). Individuals from SCD-clinical samples also exhibit neurostructural changes in MTL. In particular, it has been demonstrated that these individuals have reduced hippocampus volume (7, 21–26), although other studies did not replicate these findings (11, 27–34). Moreover, these subjects also have volume reductions and/or cortical thinning in the parahippocampus and the entorhinal cortex (28, 32, 33, 35), but again these findings have not been replicated in other studies (26, 34, 36, 37). In addition to these MTL neuroanatomical changes, there is evidence suggesting that SCD-clinical samples also display neurostructural changes that affect the frontal and parietal lobe as well as subcortical structures such as the thalamus, corona radiata and cholinergic basal nuclei (37, 38). Regarding the frontal and parietal lobe, it has been found that SCD-clinical samples have volume reductions in parietal and frontal lobe regions (21, 29), although these findings have not been replicated in other studies (34). Interestingly, direct comparison of SCD-community and SCD-clinical samples revealed more widespread structural changes in frontal, parietal, temporal (including hippocampus and parahippocampus) lobe regions and the bilateral insula in SCD-clinical samples (39, 40).

Another important factor contributing to the differences in findings is the difficulty in differentiating individuals with cognitive complaints who are undergoing normative aging from those in preclinical (41) stages of AD. In this regard, considering the recommendation by Jessen et al. (2) of the usefulness of “validated cut-off for classifying specific groups of individuals and for quantifying the severity of SCD in a research setting,” Pereiro et al. (42) recently showed that considering a cut-off point in a questionnaire to assess the severity of SCCs, in addition to the two main diagnostic SCD criteria, improves the validity of prediction of progression from SCD to MCI and/or AD dementia (42). Regarding the neuropsychological assessment of SCD, Jessen et al. (2) also pointed out that comprehensive neuropsychological test batteries that assess multiple cognitive domains, for which age, sex, and education-adjusted normative data are available, are preferable to short psychometric tests with limited diagnostic accuracy (2). It is therefore possible that cognitive examination by use of comprehensive neuropsychological test batteries may be more appropriate than short psychometric tests for detecting subtle cognitive changes that may occur in preclinical stages such as SCD.

Finally, as mentioned above, another important source of variability in the findings reported in SCD-related literature may be at least partly due to differences in the neuroimaging methods used. It has been highlighted that, although voxel-based morphometry and surface-based morphometry are the most commonly used, other imaging methods such as manual segmentation of brain structures represents 21% of studies in both SCD-community and SCD-clinical samples (13). By comparing the hippocampal findings obtained using different neuroimaging methods and reported in the SCD literature, Pini and Wennberg (13) concluded that voxel-based morphometry may be more sensitive than manual segmentation for detecting atrophy in the earliest stages of dementia and therefore that these procedures may reveal more consistent evidence regarding gray matter (GM) differences in the hippocampus, a critical region in AD dementia.

Taking all of the above considerations into account, the present study is intended to evaluate the neurocognitive and neuroanatomical changes in clinical sample of individuals who meet the two main diagnostic criteria for SCD, as proposed by the SCD-initiative (SCD-I) Working Group (1), relative to a group comprising control individuals, by extensive neuropsychological evaluation and validated sMRI procedures, respectively. The specific aims were to evaluate the following: (1) between-group differences in gray/white matter volume and cortical thickness; and (2) structural changes in the AD signature index proposed by Schwarz et al. (6). Considering previous findings, we hypothesized that, relative to the control group, individuals with SCD would display reduced volume and cortical thinning in MTL structures, parietal areas and frontal brain regions.

## 2. Materials and methods

### 2.1. Participants

The study included 98 individuals over 50 years old (73 women and 25 men), already participating in the Compostela Aging Study (CompAS) and recruited between June 2016 and January 2018. The CompAS is an ongoing longitudinal project (43) which has as its general objective the early detection and progression of cognitive impairment in patients aged + 50 years attending Primary Care Health Centers in Galicia (an autonomous community in NW Spain) with subjective cognitive complaints (SCCs). To date, the CompAS is composed of two cohorts. The first (from 2008 to 2014) included 878 individuals as eligible participants, of which 435 were excluded on the basis of the following exclusion criteria: prior diagnosis of depression or other psychiatric disturbances, according to the Diagnostic and Statistical Manual of Mental Disorders, Fifth Edition (DSM-5) criteria (44); prior diagnosis of neurological disease, including probable AD or other types of dementia, according to the National Institute of Neurological and Communicative Disorders and Stroke and the Alzheimer’s Disease and Related Disorders Association (NINCDS-ADRDA) (45) and DMS-5 criteria (44); previous brain damage or brain surgery; previous chemotherapy; prior diagnosis of diabetes type II; sensory or motor disturbances; and consumption of substances that might affect normal performance of the tasks. The second cohort is composed of 505 eligible individuals, 178 of whom were excluded according to the exclusion criteria. The participants of the current study belong to the second cohort.

Participants gave their written informed consent prior to taking part in the study. The research project was approved by the Galician Clinical Research Ethics Committee (Xunta de Galicia, Spain) and was performed in accordance with the ethical standards established in the 1964 Declaration of Helsinki (46). Ninety-two participants were right-handed, three were left-handed and three were ambidextrous, as evaluated by the Edinburgh Handedness Inventory (47). All participants had normal audition and normal or corrected-to-normal vision.

### 2.2. Neuropsychological assessment

The participants underwent clinical, neurological and neuropsychological examination conducted respectively by general practitioners, cognitive neurologists and psychologists specialized in aging and dementia. The Spanish version of the Mini Mental State Examination (MMSE) (48) was administered to all participants in order to evaluate their general cognitive functioning, and the Spanish version of the Geriatric Depression Scale (GDS-15) (49) was administered to evaluate depressive symptoms (50). Other clinical instruments frequently used to assess the early cognitive manifestations of AD and other types of dementia were also administrated. Most of these instruments have been used to diagnose impairments in several domains or cognitive processes in MCI (2, 51–54). To evaluate attentional processes, we included the Trail Making Test A (55), which assesses attentional visual-perceptive searching and perceptive-motor processing speed, and the Attention and Calculation CAMCOG-R subscale (Cambridge Cognitive Assessment-Revised) which assesses attentional control (56). In order to assess executive functioning, we used the Trail Making Test B (55), which evaluates working memory and cognitive flexibility (57), the Phonological verbal fluency test (say words starting with “p” in 1 min), which assesses working memory and inhibition (58), and the Executive Function CAMCOG-R subscale, which assesses abstract thinking and categorization. For memory processes, we used the List A Total Recall (immediate memory of words), the Long-Delay Free Recall (long term verbal memory of words) from the California Verbal Learning Test (CVLT) (59; Spanish version by 60) and the Memory CAMCOG-R subscale, which together give a joint measure composed by short delay visual memory for objects and recognition, and recent and remote memory. To evaluate language processes, we included the Boston naming test (BNT) (61), the Spanish version of the Semantic verbal fluency (animals) (58) and the Language CAMCOG-R subscale, which together provide a joint measure of oral comprehension, repetition, naming, and reading comprehension. The Lawton and Brody Index (maximum possible scoring = 8) was used to evaluate Instrumental Activities of Daily Living (IADL) (62).

### 2.3. Assessment of SCCs and diagnosis of SCD

To evaluate the severity of SCCs, we used a short Spanish version of the Questionnaire for Subjective Memory Complaints (QSMC) (63, 64). This version comprises 7 items each scored on a Likert scale ranging from 1 to 5 (maximum score 35) and was administered to participants and to a family member to assess prospective and retrospective forgetfulness, distractions and difficulties in lexical access and spatial orientation. The QSMC items were as follows: (1) “Do you forget where you left your things?”; (2) “Do you forget names of people you just met?”; (3) “Do you forget names of close relatives or friends?”; (4) “Do you often have a word on the tip of your tongue?”; (5) “Are you lost in familiar places where you have been before?”; (6) “Are you lost in unfamiliar places where you have been a few times?”; and (7) “Do you forget things you planned to do?” The reliability of this QSMC short version, tested in participants from the first cohort of the CompAS (*N* = 878) was 0.69 (Cronbach’s alpha) for patient score and 0.78 for informant scoring. The cut-off point, which corresponds to the 5% percentile of the total QSMC scoring adjusted for age, has been shown to be a valid measure of SCC severity to predict progression from SCD to MCI and dementia (predictive validity values: Sensitivity = 0.56; Specificity = 0.95; Accuracy = 0.86; NPV = 0.82) and from MCI to dementia (Sensitivity = 0.89; Specificity = 0.87; Accuracy = 0.88; NPV = 0.94) (42).

Study participants were classified according to clinical, neurological and neuropsychological data as SCD (*n* = 49) or Control (*n* = 49), at a special meeting of the research team. Participants were diagnosed as SCD when they met the two main criteria proposed by the SCD-initiative (SCD-I) Working Group (1, 2): (1) self-experienced persistent decline in cognitive capacity, especially in memory, relative to a previously normal cognitive status, which is unrelated to an acute event; and (2) normal performance in standardized cognitive tests used to classify MCI, adjusted for age and education. For the first criterion, we asked the participants if they were worried about their failures in attention and memory in the last few years, and we asked the informants for confirmation (or otherwise) of the yes/no answers. In addition, to determine whether the level of SCCs was higher than in other people of the same age, we established the 5% percentile of the total QSMC scoring (patient) adjusted for age as cut-off point. Participants who reported SCCs but did not fulfill the previous SCD criteria and did not exhibit objective cognitive impairment in the neuropsychological tests, according to norms for aged and education, were categorized as controls. Both groups were matched regarding age, gender and years of education. Demographics and between-group differences in the neuropsychological measures (calculated by the corresponding analyses) are summarized in Table 1.

**TABLE 1:** Mean values and standard deviations (SD, in brackets) of demographic and neuropsychological measures in the control (cognitively unimpaired individuals) and the subjective cognitive decline (SCD) groups.

	Control *N* = 49	SCD *N* = 49	*p* = *	Cohen’s d effect sizes
Age	65.80 (6.75)	68.12 (8.63)	0.140	0.30
Years of education	11.88 (5.26)	10.39 (5.11)	0.158	0.29
Gender (Female/Male)	38/11	35/14	0.487^a^	
GDS-15 score	2.49 (2.68)	3.63 (2.56)	0.033	0.44
**Subjective cognitive complaints**				
Patient	15.53 (2.24)	20.10 (1.99)	<0.001	2.16
Informant-caregiver	14.00 (3.27)	16.03 (3.85)	0.006	1.20
**General functioning**				
MMSE	28.53 (1.69)	28.02 (1.56)	0.123	0.31
**Attention**				
TMT-A (seconds)	51.20 (26.91)	51.76 (20.83)	0.910	0.02
CAMCOG-R (Attention and calculation)	7.69 (1.61)	7.43 (1.37)	0.382	0.17
**Executive function**				
TMT-B (seconds)	132.78 (77.85)	137.94 (63.10)	0.719	0.07
Phonological verbal fluency (letter p)	14.59 (4.84)	13.12 (5.19)	0.150	0.29
CAMCOG-R (Executive function)	21.20 (4.21)	21.24 (11.43)	0.981	0.005
**Memory**				
CVLT (Long-delay free recall)	12.04 (2.29)	11.41 (2.39)	0.184	0.27
CVLT (List A immediate total recall)	53.06 (8.24)	50.82 (9.70)	0.220	0.25
CAMCOG-R (Memory)	22.61 (2.33)	21.74 (2.50)	0.075	0.36
**Language**				
BNT	50.82 (6.94)	50.33 (6.78)	0.725	0.07
Semantic verbal fluency (Animals)	20.10 (5.27)	17.39 (5.14)	0.011	0.52
CAMCOG-R (Language)	27.02 (2.00)	26.27 (2.36)	0.090	0.34
**Instrumental activities of daily living**				
IADL (Lawton and Brody index)	7.89 (0.36)	7.56 (0.91)	0.015	0.48

Two sample *t*-test; *p < 0.05. GDS-15, geriatric depression scale; MMSE, mini-mental state examination; TMT-A/B, trail making test (version A/B); CVLT, California verbal learning test; CAMCOG-R, Cambridge cognitive examination; BNT, Boston naming test; IADL, Lawton and Brody index; SCD, subjective cognitive decline. *a* = Chi squared test.

### 2.4. MRI acquisition and data analysis

For structural MRI analysis, a sagittal T1-weighted 3D-MPRAGE sequence (repetition time/echo time = 7.45 ms/3.40 ms, flip angle = 8°; 180 slices, voxel size = 1 × 1 × 1 mm, field of view = 240 × 240 mm^2^, matrix size = 240 × 240 mm) was acquired with a Philips 3T Achieva scanner (Philips Medical System, Best, The Netherlands), in the University Hospital Complex, Santiago de Compostela, Galicia (Spain).

In order to evaluate differences in gray matter (GM) and white matter (WM) volume between groups, a voxel-based morphometry analysis was conducted in Matlab R2016a by using the Computational Anatomy Toolbox^1^ implemented in the Statistical Parametric Mapping software (SPM12^2^). After visual quality control, T1-weighted images were manually reoriented to the anterior-posterior commissure, segmented in GM and WM tissues (65) and normalized to the Montreal Neurological Institute space using a customized template built with the DARTEL toolbox (66). Normalized and modulated GM/WM images were then spatially smoothed with a Gaussian kernel of 8 mm Full Width at Half Maximum (FWHM).

Statistical analyses were conducted using the General Linear Model (GLM) approach, and between-group analysis was performed *via* two sample *t*-tests including the total intracranial volume and the GDS scores as covariates (the SCD group displayed higher depressive symptoms, but below the 5–7 cut-off score for mild depression, see Table 1). Statistical analyses were conducted considering the whole brain as the volume of interest. Finally, voxel-wise permutation testing (10,000 permutations) was conducted by the Threshold Free Cluster Enhancement (TFCE) method with the TFCE toolbox.^3^ Results were considered significant at *p* < 0.05 Family-Wise Error (FWE).

Cortical thickness differences were evaluated by surface-based morphometry analysis, with FreeSurfer 6.0 software.^4^ The automated default preprocessing pipeline was used for cortical reconstruction and volumetric segmentation (67, 68). The preprocessing pipeline included motion correction, skull stripping, transformation into the Talairach space, segmentation of cortical and subcortical GM/WM volumetric structures, intensity normalization, tessellation of the boundary between GM and WM, and topology correction. A quality control protocol was conducted over the FreeSurfer segmentations with the Freeview program. FreeSurfer segmentations were visually inspected on a slice-by-slice basis by an experienced technician, to enhance the reliability of the cortical thickness measurements. Pial surface misplacement errors that included meninges and the skull were manually corrected in all subjects. Moreover, erroneous white matter segmentation due to intensity normalization errors was fixed in seventy-four participants by using control points. All manual editions were conducted following the technical instructions included in the Freeview Guide.^5^ Final segmentations were supervised by a senior researcher (SGA). Between-group analysis was performed by a GLM including the GDS scores as covariate and applying a Monte Carlo simulation to correct for multiple comparisons with 10,000 iterations, a cluster-forming threshold set at *p* < 0.005 and a smoothing kernel of 15 mm FWHM. Additionally, *p*-values were adjusted for both hemispheres applying the Bonferroni correction, and results were considered significant at *p* < 0.05.

A follow-up ROI analysis was performed over MTL. Hippocampal subfields were automatically segmented with FreeSurfer (69). The following volume measurements were visually inspected before being exported: whole hippocampus including head, body and tail; the parasubiculum; the head and body of the presubiculum, subiculum, CA1, CA3 (CA2 is included in CA3), CA4, granulate cell of the molecular layer of dentate gyrus, hippocampal molecular layer, hippocampal fissure, fimbria and the hippocampus-amygdala transition area. The entorhinal cortex and parahippocampal gyrus volume and all hippocampal subfield volume measurements were adjusted using the estimated total intracranial volume (eTIV) by the residual approach: adjusted_volume = volume_observed–b × (eTIV–mean_eTIV), where mean_eTIV is the average eTIV of all subjects, and b is the coefficient of regression between the observed volume and the eTIV. In comparison with other approaches, the residual adjustment method proved optimal for discriminating between Control subjects and individuals with AD dementia and also between individuals with MCI and patients with AD (70). Between-group analysis was performed using a multivariate GLM including Group as the fixed factor, the adjusted volume measures as dependent variables and the GDS scores as covariate. The Bonferroni method was used to correct for multiple comparisons, and the significance level was set at *p* < 0.05.

Finally, the AD signature index was computed by averaging the thickness estimates from the entorhinal cortex, inferior temporal gyrus, middle temporal gyrus, inferior parietal lobe, fusiform gyrus and precuneus (6). Between-group differences in the AD signature index were evaluated using a univariate GLM including the Group as fixed factor, the AD signature index as a dependent variable and the GDS scores as covariate. Results were considered significant at *p* < 0.05.

## 3. Results

### 3.1. Between group analysis

The demographic data and results of the neuropsychological examinations are summarized in Table 1. There were no significant differences between the groups regarding age, years of education or gender, but, as expected, there were significant differences in the SCCs. Moreover, there were no significant differences in any cognitive test except in one neuropsychological language test (Semantic Verbal Fluency test). Relative to IADL, both groups scored next to the maximum, but the control group scores were significantly higher than those of the SCD group.

**FIGURE 1:**
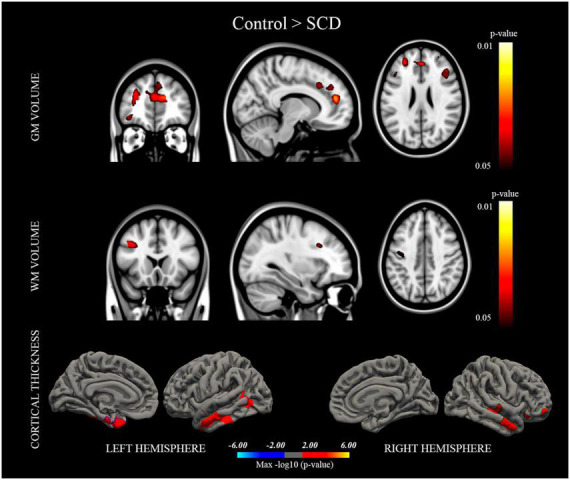
Brain regions in which the control group displayed significantly higher gray matter/white matter (GM/WM) volume and cortical thickness than the subjective cognitive decline (SCD) group. Results were considered statistically significant at *p* < 0.05.

Volume and cortical thickness data are summarized in Table 2 and illustrated in Figure 1. Relative to control subjects, individuals with SCD displayed significant reductions in GM volume in the triangular part of the inferior frontal gyrus, the orbital part of the middle frontal gyrus, the superior and middle frontal gyrus and the superior medial frontal gyrus of both hemispheres, the orbital part of the medial frontal gyrus of the left hemisphere, the bilateral anterior and the right middle cingulate cortex, and the left precentral/postcentral gyrus. Moreover, relative to the control group, the SCD group displayed reductions in WM volume in the left triangular part of the inferior frontal gyrus and the left precentral/postcentral gyrus, as well as cortical thinning in the left inferior and the right middle temporal gyrus, the left entorhinal cortex and the right lateral orbitofrontal cortex.

**TABLE 2:** Brain regions showing significant differences in gray matter (GM) and/or white matter (WM) volume and cortical thickness in the between-group analyses.

	Combined peak-cluster level								
	Brain region	Cluster size	L/R	MNI coordinates			TFCE-FWE *p*-value		
				*X*	*Y*	*Z*			
**Gray matter Control > SCD**									
Volume	Anterior cingulate cortex	7390	R	16	46	19	0.018		
	Anterior cingulate cortex*		L	1	42	22	0.025		
	Midcingulate cortex		R	10	23	36	0.037		
	Superior medial frontal gyrus		R	7	42	37	0.038		
	Superior frontal gyrus		R	13	40	33	0.038		
	Middle frontal gyrus		R	27	34	36	0.038		
	Middle frontal gyrus	6792	L	−28	42	20	0.030		
	Middle frontal gyrus (orbital part)		L	−36	45	−8	0.037		
	Superior frontal gyrus		L	−27	54	2	0.039		
	Medial frontal gyrus (orbital part)		L	−14	56	−2	0.041		
	Superior medial frontal gyrus		L	−14	60	10	0.043		
	Inferior frontal gyrus (triangular part)	1519	R	41	22	15	0.033		
	Postcentral gyrus	1650	L	−54	−5	42	0.034		
	Precentral gyrus		L	−44	5	42	0.046		
	Middle frontal gyrus (orbital part)	569	R	33	50	−2	0.041		
	Inferior frontal gyrus (triangular part)	491	L	−47	16	31	0.041		
**White matter Control > SCD**									
	Inferior frontal gyrus (triangular part)	1142	L	−37	18	32	0.028		
	Postcentral gyrus	718	L	−42	−13	39	0.047		
	Precentral gyrus		L	−49	−5	49	0.048		
	**Brain region**	**Cluster size (mm^2^)**	**L/R**	**MNI coordinates**			**Max-log10(*p*-value)**	**CWp**	**Cohen’s D**
				***X***	***Y***	***Z***			
**Control > SCD**									
Thickness	Inferior temporal gyrus	1506.29	L	−46.8	−36.1	−23.6	4.59	0.0002	1.06
		842.93	L	−50	−63.8	−3.6	4.84	0.009	1.03
	Entorhinal cortex	848.82	L	−26.5	−9.3	−33.7	3.62	0.008	1.03
	Middle temporal gyrus	1236.69	R	57.4	−1.2	−28.1	5.21	0.0004	1.02
	Lateral orbitofrontal	708.99	R	30.8	33.5	−7.8	3.94	0.029	0.99

L/R, left or right hemisphere; MNI, montreal neurological institute coordinates; TFCE, threshold free cluster enhancement; FWE, family wise error; Max-log10(*p*-value), maximum-log10(*p*-value) at each cluster; CWP, clusterwise *p*-value; Cohen’s D, effect sizes. *According to the Automated Anatomical Labeling (AAL) atlas, this coordinate (*X* = 1; *Y* = 42; *Z* = 22) is located in the left anterior cingulate cortex even though the x-coordinate is positive. However, other left coordinates of the anterior cingulate are also significant within this cluster.

The follow-up ROI analysis revealed that, relative to controls, individuals with SCD displayed a significantly reduced volume in the left hippocampus tail, left head of the subiculum, right fimbria and right parahippocampal gyrus, as well as significant thinning of the left entorhinal cortex and the right parahippocampal gyrus (see Table 3).

**TABLE 3:** Mean values and standard deviations (SD in brackets) of the adjusted hippocampal subfields measures and the surrounding medial temporal lobe areas.

Brain region			Control *N* = 49		SCD *N* = 49		*p*		Cohen’s D	
			Left	Right	Left	Right	Left	Right	Left	Right
**Volume (mm^3^)**										
Hippocampus	Whole hippocampus		3106.63 (293.33)	3199.74 (297.82)	3024.21 (344.62)	3169.10 (346.94)	0.172	0.543	0.26	0.10
	Hippocampus	Head	1523.10 (158.68)	1600.42 (164.84)	1497.46 (197.21)	1590.13 (198.96)	0.435	0.715	0.14	0.06
		Body	1068.03 (106.79)	1081.15 (96.91)	1040.69 (118.82)	1068.35 (118.29)	0.205	0.455	0.24	0.12
		Tail	515.49 (60.80)	518.17 (69.59)	486.06 (66.79)	510.61 (58.64)	**0.016**	0.442	**0.46**	0.12
	Presubiculum	Head	133.50 (17.74)	128.09 (17.84)	126.89 (18.10)	126.68 (18.20)	0.069	0.679	0.37	0.08
		Body	148.44 (25.57)	135.30 (23.42)	141.50 (21.93)	130.09 (20.69)	0.179	0.247	0.29	0.24
	Subiculum	Head	177.57 (23.20)	178.72 (22.09)	167.83 (25.40)	175.75 (27.64)	**0.048**	0.516	**0.40**	0.12
		Body	223.61 (24.29)	221.21 (23.02)	215.47 (28.49)	217.39 (25.85)	0.134	0.328	0.31	0.16
	Parasubiculum		63.91 (14.44)	61.07 (11.17)	60.22 (13.88)	59.16 (13.85)	0.254	0.466	0.26	0.15
	CA1	Head	454.03 (48.14)	485.54 (49.74)	454.17 (60.99)	486.57 (62.94)	0.957	0.993	0.003	0.02
		Body	108.71 (19.89)	118.03 (17.67)	109.88 (22.92)	120.34 (22.38)	0.894	0.694	0.06	22.38
	CA3*	Head	105.03 (16.12)	116.98 (16.06)	106.23 (19.02)	117.40 (18.80)	0.831	0.958	0.07	0.02
		Body	75.97 (13.08)	86.94 (11.83)	77.85 (14.95)	89.90 (16.23)	0.661	0.385	0.13	0.21
	CA4	Head	110.57 (12.76)	119.67 (13.68)	110.73 (17.06)	119.49 (15.85)	0.919	0.872	0.01	0.01
		Body	107.70 (12.14)	112.29 (11.86)	105.63 (13.61)	113.46 (13.89)	0.401	0.695	0.16	0.09
	GC.ML.DG	Head	131.79 (16.31)	143.44 (17.12)	131.89 (21.34)	143.41 (20.34)	0.902	0.905	0.005	0.002
		Body	121.83 (14.57)	125.23 (13.33)	118.04 (15.16)	125.48 (15.37)	0.200	0.983	0.26	0.02
	Molecular layer HP	Head	294.13 (30.47)	309.50 (32.44)	287.92 (38.42)	306.59 (39.33)	0.350	0.631	0.18	0.08
		Body	201.90 (22.95)	207.35 (21.35)	195.00 (25.23)	204.63 (24.79)	0.137	0.483	0.29	0.12
	Hippocampal fissure		152.12 (25.10)	168.58 (22.16)	152.23 (25.32)	176.38 (29.68)	0.890	0.145	0.004	0.30
	Fimbria		79.87 (16.88)	74.80 (18.20)	77.31 (19.13)	67.08 (19.28)	0.422	**0.032**	0.14	**0.41**
	HATA		52.57 (10.16)	57.40 (10.42)	51.58 (9.65)	55.08 (11.25)	0.494	0.222	0.10	0.21
PHG			1930.67 (263.16)	1845.76 (234.86)	1880.51 (287.25)	1728.77 (190.88)	0.509	**0.005**	0.19	**0.55**
Entorhinal C.			1518.16 (260.16)	1560.71 (217.92)	1524.64 (272.26)	1569.70 (277.70)	0.865	0.699	0.02	0.04
**Thickness (mm)**										
PHG			2.63 (0.24)	2.56 (0.23)	2.58 (0.26)	2.48 (0.15)	0.354	**0.044**	0.20	**0.41**
Entorhinal C.			3.18 (0.27)	3.14 (0.32)	3.05 (0.34)	3.12 (0.35)	**0.009**	0.668	**0.42**	0.06

SCD, subjective cognitive decline; PHG, parahippocampal gyrus; Entorhinal C, entorhinal cortex; CA, cornu ammonis; GC.ML.DG, granule cell of the molecular layer of dentate gyrus; Molecular layer HP, molecular layer of the hippocampus; HATA, hippocampus-amygdala-transition-area; Cohen’s D, effect sizes. *CA2 is included in the CA3 subfield. Regions in which the analyses revealed statistically significant between-group differences for the volume and thickness are highlighted in bold.

Moreover, between-groups comparisons revealed that the AD signature index was significantly lower (*p* = 0.005) in the SCD group (mean: 2.53; SD: 0.10) than in the control group (mean: 2.57; SD: 0.10).

## 4. Discussion

The present study aimed to evaluate neuroanatomical differences between individuals with SCD and control subjects. The results revealed that individuals with SCD showed subtle structural changes in similar brain regions to those observed in amnestic MCI and AD dementia including MTL, frontal cortex and parietal regions (7–9). These neurostructural changes are located in some regions with important functional roles in several cognitive domains such as executive function, attention, episodic memory and language.

The cognitive performance was generally similar in both groups. However, the SCD group performed the semantic verbal fluency test less well than the control group. This result is consistent with previous evidence of poorer performance in semantic verbal fluency measures in people with SCD relative to control subjects (71). Moreover, the absence of extensive cognitive differences between the two groups was not unexpected. SCD has, by definition, been proposed as a possible preclinical stage of AD in which people display subjective cognitive complaints (SCCs), but in whom objective evidence of cognitive impairment is not usually detected by neuropsychological assessment (1). As Jessen et al. (2) pointed out, neuropsychological assessments for screening SCD are usually conducted using short psychometric tests with limited diagnostic accuracy. However, the use of comprehensive neuropsychological test batteries that assess multiple cognitive domains, and for which age, sex, and education-adjusted normative data are available (2) is preferable. We therefore hypothesized that the exhaustive cognitive assessment conducted in the present study would successfully detect the subtle cognitive differences revealed by the semantic verbal fluency test performance in the SCD group.

The need for accurate detection of neuroanatomical changes before the onset of extensive cognitive deficits in individuals with SCD highlights the importance of using techniques with high spatial resolution (e.g., sMRI) to locate early neurostructural changes in people with SCCs who are at risk of developing AD dementia. This, in turn, could be of interest in regard to implementing future non-pharmacological interventions aimed at preventing cognitive impairment. This is of particular importance if we take into account previous studies demonstrating, for example, that people with subjective memory complaints, but not objective impairment, are two times more likely to develop dementia than individuals without subjective memory complaints (5).

Several of the present study findings suggest that SCD may be related to neurophysiopathological changes that occur in AD. First, SCD participants displayed reduced cortical thickness in the AD signature index. This AD neuroimaging marker captures the cortical thinning of AD vulnerable regions and is therefore consistent with the brain atrophy characteristic of the early stages of AD onward (72). Atrophy in those regions including the AD signature is observed in MCI patients with presence of beta-amyloid deposits (6, 73, 74). Therefore, the results suggest that the psychometric criterion of SCC severity proposed by Pereiro et al. (42) for diagnosis of SCD may be appropriate for early detection of AD, consistent with the prognostic value of progression from preclinical and prodromal stages to AD dementia (42).

The present findings have indeed shown a pattern of neurostructural changes in the SCD group congruent with that described for AD (72). Within the MTL, brain changes were located in the hippocampal tail, the head of subiculum, the entorhinal cortex of the left hemisphere and also in the fimbria and the parahippocampal gyrus of the right hemisphere. Neurodegeneration of MTL is a characteristic feature in the etiology of AD dementia. According to Braak and Braak (72), the earliest presence of neurofibrillary tangle deposition takes place in MTL areas, including the entorhinal cortex, hippocampus and the parahippocampal gyrus. Moreover, MTL plays an essential role in episodic memory, the cognitive domain most affected in AD dementia. Previous studies aimed at evaluating structural changes in the hippocampus and its subfields revealed atrophy in CA1 and the subiculum subfields of individuals with SCD or AD dementia (7, 33). In addition to the hippocampus and the subiculum, structural changes also affected the entorhinal cortex, and the parahippocampal gyrus. Neurostructural changes in both MTL areas were observed in SCD (33) and in MCI and AD dementia (75, 76); in addition, these changes may represent a biomarker of progression in AD (77, 78).

Beyond the MTL, SCD individuals displayed cortical thinning of the left inferior and the right middle temporal gyrus. Synaptic loss in the inferior temporal gyrus has been demonstrated in amnestic MCI (79), and neurodegeneration in these regions is considered a good predictor of decline in conversion to AD dementia (80). Convit et al. (80) demonstrated that, together with some occipito-temporal areas (fusiform gyrus), the inferior and middle temporal gyrus are the neocortical regions that are first affected in the progression toward AD dementia. These researchers observed that structural changes in MTL, occipito-temporal areas and temporal regions may predict the decline of control subjects and individuals with MCI toward AD dementia.

Thus, in the light of these findings regarding the temporal lobe, the present results seem to indicate that SCD is associated with structural changes in regions of critical importance in AD etiology and with an essential role in episodic memory.

Regarding the frontal lobe, individuals with SCD displayed reductions in GM volume in the triangular part of the inferior frontal gyrus, the orbital part of the middle frontal gyrus, the superior and middle frontal gyrus and the superior medial frontal gyrus of both hemispheres, as well as in the orbital part of the medial frontal gyrus and the precentral gyrus of the left hemisphere. In addition, the SCD group displayed cortical thinning in the right lateral orbitofrontal cortex. These results are consistent with the findings of previous studies reporting structural changes in the frontal lobe of individuals with SCD (9, 29, 39).

We also observed structural changes in the bilateral anterior and the right middle cingulate cortex in individuals with SCD. Prior evidence suggests that, while the midcingulate cortex is functionally related to successful episodic memory retrieval (81), the anterior cingulate and the dorsolateral prefrontal cortex support retrieval monitoring, a control process that evaluates retrieval outcomes in relation to behavioral goals (82). Therefore, considering the functional relationship between frontal lobe and cognitive processes, such as attention, executive functioning and language (83), and the involvement of the cingulate system in the aforementioned cognitive control processes, the present results suggest that individuals with SCD display subtle changes that may affect the structure of brain networks supporting attention, executive function, language, successful episodic memory retrieval, and cognitive control processes (e.g., retrieval monitoring).

The SCD group also displayed reductions in GM in the left postcentral gyrus. Parietal lobe atrophy has been demonstrated in subjects with amnestic MCI or AD dementia (84, 85) and also in earlier stages, such as SCD (86). Considering that parietal lobe is highly interconnected with several brain areas, structural changes in this lobe may be related to the progressive neuropsychological decline in several cognitive domains (e.g., attention, memory, language, and executive function) commonly displayed by patients along the AD continuum (87).

There is evidence to suggest that, despite some common features, individuals recruited from memory clinics and who reported concerns about their cognition (SCD-clinical samples) displayed more widespread neurostructural changes involving frontal, parietal, temporal (including hippocampus and parahippocampus) lobe regions and the insula, relative to population-based cohorts (SCD-community samples) (39, 40). Structural changes in SCD-clinical samples have been attributed to comorbid mood disorder symptomatology that may be related to a more complex neurodegenerative pattern than that observed in SCD-community samples (13).

However, we evaluated a SCD-clinical sample recruited from Primary Care Health Centers, and none of the participants had prior diagnosis of any psychiatric disorder. Nonetheless, the SCD group displayed higher levels of depressive symptomatology. Depressive symptoms are commonly observed in individuals with SCD, and it has been shown that depression and SCD are independently associated with the risk of developing MCI and dementia, with hazard ratios of 1.4 and 2.0, respectively (88). Importantly, the co-occurrence of SCD and depression was associated with the highest risk (hazard ratio = 2.8) of developing a neurocognitive disorder within 7.2 years of follow-up (compared to 12.2 years in participants without depression or SCD) (88). These findings were recently confirmed in a nationwide longitudinal study (89). Therefore, considering that neither of the two groups had scores compatible with mild depression and that depressive symptomatology was included as a covariate in the sMRI analyses, the neurostructural differences observed in the SCD group could not be explained either by differences in the study setting or comorbid effects of mood disorder symptomatology in the SCD group. Thus, one possible explanation for the neurostructural changes displayed by the SCD group in frontal brain areas and also in other regions of critical importance in AD dementia (especially those included in the AD signature) may be related to structural changes occurring during progression from SCD to prodromal stages (i.e., MCI) and AD dementia.

The present study has some limitations that are worth noting. Its cross-sectional nature, together with the relatively small sample size, limits evaluation of the clinical trajectory and the neuroanatomical changes that may take place in a hypothetical progression from SCD toward MCI or AD dementia. Moreover, future studies should consider examining other AD biomarkers (e.g., CSF, PET or blood-based) that may reveal where participants are in the AD continuum. In addition, despite the absence of significant differences between groups regarding age and gender, only 25% of the study population were men. Future studies should evaluate the neuroanatomical and cognitive differences in larger and better matched samples with an equal proportion of males and females within each group to enable generalization of the results.

## 5. Conclusion

In summary, application of the diagnostic criterion of SCD using the levels of SCC severity proposed by Pereiro et al. (42) revealed that individuals with SCD have an objective, measurable pattern of subtle neurostructural and neurocognitive changes consistent with those reported in prodromal and clinical stages of the AD continuum. Structural changes were located in MTL, frontal and parietal areas with a critical role in several cognitive domains affected in AD dementia, including executive control, attention, episodic memory and language. Thus, the results emphasize the need to focus future research on preclinical stages (i.e., SCD) of the AD continuum to assess the prognostic value of the structural and neurocognitive changes observed. The early detection of these neurostructural changes before the onset of clinical symptoms may have important implications for the application of pharmacological and/or non-pharmacological therapies to prevent cognitive impairment in those individuals at risk of progressing toward AD dementia.

## Data availability statement

The raw data supporting the conclusions of this article will be made available by the authors, without undue reservation.

## Ethics statement

The studies involving human participants were reviewed and approved by Galician Clinical Research Ethics Committee, Xunta de Galicia. The patients/participants provided their written informed consent to participate in this study.

## Author contributions

MR-F: methodology, formal analysis, writing – original draft, visualization, and manuscript revision. ML and SG-Á: conceptualization, methodology, investigation, visualization, supervision, project administration, and manuscript revision. MZ and FD: conceptualization, investigation, resources, supervision, project administration, funding acquisition, and manuscript revision. CL-S: investigation, formal analysis, and manuscript revision. AP: conceptualization, methodology, investigation, formal analysis, project administration, and manuscript revision. All authors contributed to the article and approved the submitted version.

## Footnotes

1
http://www.neuro.uni-jena.de/cat/

2
https://www.fil.ion.ucl.ac.uk/spm/

3
http://www.neuro.uni-jena.de/tfce/

4
http://surfer.nmr.mgh.harvard.edu/

5
https://surfer.nmr.mgh.harvard.edu/fswiki/FreeviewGuide
